# Cardioprotection with Intralipid During Coronary Artery Bypass Grafting Surgery on Cardiopulmonary Bypass: A Randomized Clinical Trial

**DOI:** 10.1007/s10557-024-07594-w

**Published:** 2024-06-12

**Authors:** Nkanyiso Hadebe, Martin Cour, Aqeela Imamdin, Tarra Petersen, Timothy Pennel, Jacques Scherman, Jane Snowball, Mpiko Ntsekhe, Peter Zilla, Justiaan Swanevelder, Sandrine Lecour

**Affiliations:** 1 Cardioprotection Group, Faculty of Health Sciences, Cape Heart Institute, University of Cape Town, Anzio Road, Cape Town, 7925 Observatory South Africa; 2 Department of Anaesthesia, Faculty of Health Sciences, University of Cape Town, Cape Town, South Africa; 3 Chris Barnard Division of Cardiothoracic Surgery, University of Cape Town, Cape Town, South Africa; 4 Division of Cardiology, Faculty of Health Sciences, University of Cape Town, Cape Town, South Africa

**Keywords:** Ischemia–reperfusion injury, Intralipid, Cardioprotection, Coronary artery bypass grafting

## Abstract

**Purpose:**

Coronary artery bypass grafting (CABG) on cardiopulmonary bypass (CPB) is associated with myocardial ischemia–reperfusion injury (IRI), which may limit the benefit of the surgery. Both experimental and clinical studies suggest that Intralipid, a lipid emulsion commonly used for parenteral nutrition, can limit myocardial IRI. We therefore aimed to investigate whether Intralipid administered at reperfusion can reduce myocardial IRI in patients undergoing CABG on CPB.

**Methods:**

We conducted a randomized, double-blind, pilot trial in which 29 adult patients scheduled for CABG were randomly assigned (on a 1:1 basis) to receive either 1.5 ml/kg Intralipid 20% or Ringer’s Lactate 3 min before aortic cross unclamping. The primary endpoint was the 72-h area under the curve (AUC) for troponin I.

**Results:**

Of the 29 patients randomized, 26 were included in the study (two withdrew consent and one was excluded before surgery). The 72-h AUC for troponin I did not significantly differ between the control and Intralipid group (546437 ± 205518 versus 487561 ± 115724 arbitrary units, respectively; *P* = 0.804). Other outcomes (including 72-h AUC for CK-MB, C-reactive protein, need for defibrillation, time to extubation, length of ICU and hospital stay, and serious adverse events) were similar between the two groups.

**Conclusion:**

In patients undergoing CABG on CPB, Intralipid did not limit myocardial IRI compared to placebo.

**Trial Registration:**

ClinicalTrials.gov Identifier: NCT02807727 (registration date: 16 June 2016).

## Introduction

Coronary heart disease is the primary cause of death worldwide, affecting both developed and developing countries [1]. Despite the improvement in the management of patients suffering from ischemic heart disease in the past 30 years, the outcome of these patients is unfortunately still limited, at least in part, due to myocardial ischemia–reperfusion (IRI) injury [2]. The delineation of a novel therapy that may limit IRI is therefore key to improve mortality and morbidity in these patients.

In this regard, Intralipid, a lipid emulsion made from soybean plant and commonly used in parenteral nutrition [3] or as a vehicle for multiple lipid soluble drugs, has shown great promise to limit IRI in both the preclinical setting and in small proof-of-concept clinical trials [4–12]. In ex vivo or in vivo experimental conditions of IRI in rodents, Intralipid reduced infarct size and improved cardiac hemodynamic functions, an effect that may involve the activation of key components of the Survivor Activating Factor Enhancement (SAFE) pathway, such as the signal transducer and activator of transcription 3 (STAT3) [4–7, 13, 14]. In humans, proof-of-concept randomized trials suggest that Intralipid given during cardiac surgery (off-pump coronary artery bypass graft (CABG) surgery or valve replacement surgery) may decrease biomarkers of myocardial IRI via mechanisms that remain elusive [8, 10, 11]. Despite this, to date, there is no perioperative trial outcome evidence to recommend any interventions that target IRI in patients undergoing on-pump CABG surgery.

In the present study, we therefore aimed to investigate whether Intralipid, administered at reperfusion, would reduce myocardial IRI in patients undergoing CABG on cardiopulmonary bypass (CPB).

## Methods

### Study Design

The CREW-I study (*Cardiac REperfusion With Intralipid at reperfusion study*) was an academic, monocentric, prospective, randomized, double-blind, pilot trial conducted at the Groote Schuur Hospital in Cape Town, South Africa. The trial was authorized by the *Human Research Ethics Council* (HREC) of the University of Cape Town (number: 806/2014) and by the *Medicines Control Council* (MCC) of South Africa (number: 20150807). The trial was registered in ClinicalTrials.gov before the start of the study (registration number: NCT02807727) on June 16, 2016. The trial was conducted according to the requirements of the Declaration of Helsinki. Written informed consent was obtained from all participants.

### Patients

Consecutive patients scheduled for first time elective isolated CABG on CPB were screened for eligibility. Patients were eligible for inclusion if they were aged between 18 and 65 years, had left ventricular ejection fraction greater than 40% and body mass index between 21 and 35 kg/m^2^. Exclusion criteria were diabetes mellitus, creatinine > 200 μmol/l, myocardial infarction within the previous 2 weeks, inotropic support prior to surgery, contraindications to Intralipid (including previous hypertriglyceridemia pancreatitis, plasma triglyceride levels > 5.7 mmol/l, egg, peanut, and soybean allergy), treatment with Glibenclamide or Nicorandil (which may interfere with cardioprotection) [15], and participation in another interventional trial within 30 days.

### Peri-operative Procedure

The decision to maintain chronic treatments (e.g., statins, beta-blockers, etc.) prior to surgery was left to the discretion of the physicians in charge and followed international guidelines. Surgery for this study was performed by two experienced surgeons using a similar technique regarding bypass management, cardioplegia, and selection of grafts; they were blinded to the treatment allocation. Induction of anesthesia consisted of intravenous sufentanil (1 µg/kg), etomidate (0.2 mg/kg), rocuronium (0.6 mg/kg). Anesthesia was maintained with isoflurane (0.6–1.8%). After median sternotomy and systemic heparinization, the ascending aorta and right atrium and were cannulated to establish CPB. After cross clamping, arrest was achieved with blood-based St. Thomas’ Hospital cardioplegic solution at mild hypothermia (34 °C). After surgery, patients were transferred to intensive care unit (ICU) and extubated at the earliest clinically appropriate time.

### Experimental Protocol

**Fig. 1:**
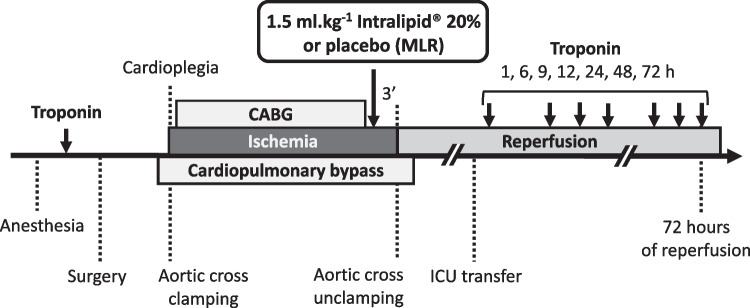
Experimental protocol of the study. MLR: Modified Lactate’s Ringer; ICU: intensive care unit

The experimental protocol is summarized in Fig. 1. Patients who met the enrollment criteria were randomized on a 1:1 basis to either control or Intralipid group using a table of random numbers generated by the study statistician. A pre-compiled study folder was assigned to each patient. The folder had a sequential randomization number together with a barcode generated by a software package (R Studio, Inc. Austria). The barcoded adhesive stickers were used for all study-related investigational procedures, including patient allocation for the issue of study drug, blood samples for analysis, and tissues for storage.

Three minutes prior to aortic unclamping (i.e., before myocardial reperfusion), patients received, via central venous catheter, either a rapid infusion of 1.5 ml/kg Intralipid 20% (Fresenius-Kabi, Port Elizabeth, South Africa) for the Intralipid group or 1.5 ml/kg of modified Ringer’s Lactate (Fresenius-Kabi, Port Elizabeth, South Africa) for the Control group, using a concealed administration set and opaque syringe. The dose of Intralipid and timing for administration of the drug was extrapolated from experimental data that showed potent protection [5]; we also considered the pharmacokinetic effect of the CBP circuit. The study duration was limited to the hospital length of stay.

### Outcomes

The primary outcome was the mean geometric area under the 72-h curve (AUC) for cardiac troponin I (cTnI) release.

Secondary outcomes included the 72-h AUC of creatine kinase-myocardial band (CK-MB), need for defibrillation, time to extubation, length of ICU and hospital stay, and serious adverse events occurring during hospitalization (death and any peri- and post-intervention complications).

### Echocardiography and Biochemical Analyses

Left ventricular ejection fraction (LVEF) was assessed in patients before inclusion by means of ventriculography. LEVF was also assessed after induction of anesthesia using transesophageal echo.

Post-enrolment, a lipid profile including plasma levels of triglycerides, total-cholesterol, low-density lipoprotein (LDL), and high-density lipoprotein (HDL) cholesterol was obtained for each patient.

Blood samples for the analysis of cTnI and CK-MB were drawn after induction of anesthesia and 1, 6, 9, 12, 24, 48, and 72 h after unclamping the aorta. C-reactive protein (CRP) was measured at 24 h after surgery to assess a potential effect of Intralipid on inflammation.

### Statistical Analysis

Based on experimental studies on Intralipid-induced cardioprotection [4–7] and a clinical trial on remote ischemic conditioning in patients undergoing CABG [16], we expected a 40% reduction of the 72 h AUC of cTnI with Intralipid. Considering a statistical power of 90% and an alfa of 0.05 (two-tailed test), the estimated number of patients per group was 15 after adjustment for withdrawals.

Continuous data are presented as median and 1st and 3rd interquartile or mean ± SEM, according to the distribution of the values. Categorial data are presented as number (percentage). Continuous variables were compared using the Wilcoxon rank-sum test or the Student’s *t* test, and the Chi-squared, or the Fisher exact test was used for proportions.

The primary endpoint was assessed by the geometric mean difference of the AUC for the cTnI concentration in serum over 72 h (sampled at 1, 6, 12, 24, 48, and 72 h), calculated according to the trapezoid rule, and compared using an unpaired *t*-test. The same method was used to compare the AUC for CK-MB. Repeated measures of cTnI and CK-MB were also compared using two-way ANOVA (group, time).

Statistical analyses were performed using GraphPad Prism 9 (version 9.4.1, La Jolla, USA). A 2-sided *P* value of 0.05 or less was considered statistically significant.

## Results

### Clinical Trial

**Fig. 2:**
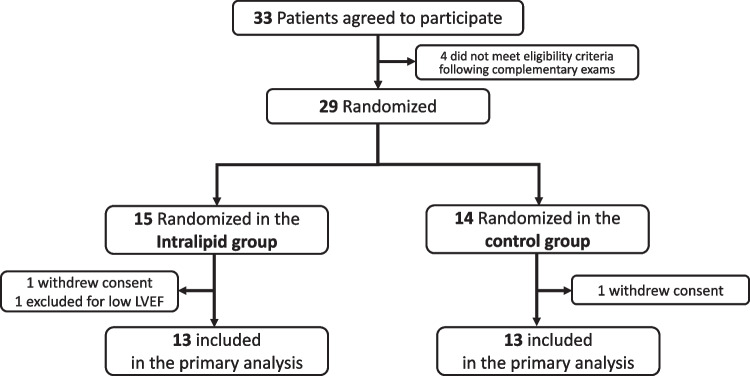
Flow chart of the study. LEVF: Left ventricle ejection fraction

From 19 June 2017 to 17 February 2020, 33 patients scheduled for first-time elective isolated CABG on CPB agreed to participate (Fig. 2). After additional examination (lipid profile, echocardiography, etc.), 29 patients were randomized (15 in the Intralipid group and 14 in the Control group). One patient in each group withdrew consent before surgery. One patient was excluded because of low left ventricular ejection fraction after induction of anesthesia. Thus, 13 patients per group were included in the analysis (Fig. 2).

No significant differences were observed between the two groups at baseline, except for distribution of cholesterol sub-types, with a significantly lower HDL cholesterol, in the Intralipid group (Table 1). None of the patients had an history of percutaneous coronary intervention. The cross-clamp time and the number grafts did not differ between the 2 groups (Table 2).

**Table 1:** Patient characteristics

	Control group (*n* = 13)	Intralipid group (*n* = 13)	*P* value
Age	55 (52–66)	59 (55–64)	0.810
Male sex	11 (85%)	9 (69%)	0.640
Race or ethnic group			0.510
Mixed*	9 (69%)	9 (69%)	
Black	1 (8%)	0 (0%)	
Caucasian	1 (8%)	3 (23%)	
Indian	2 (15%)	1 (8%)	
BMI (kg/m^2^)	28 (23–29)	25 (24–27)	0.138
Comorbidities			
Hypertension	10 (77%)	12 (92%)	0.280
Hypercholesterolemia	1 (8%)	3 (23%)	0.280
Smoking	8 (62%)	8 (62%)	> 0.99
Concomitant medications			
Aspirin	10 (77%)	11 (85%)	0.620
Clopidogrel	1 (8%)	4 (31%)	0.140
β-blockers	10 (77%)	12 (92%)	0.280
Statin	10 (77%)	12 (92%)	0.280
Diuretics	3 (23%)	1 (8%)	0.280
ACEI/ARB	8 (62%)	9 (69%)	0.680
Calcium antagonist	2 (15%)	3 (23%)	0.620
Echocardiography			
LV ejection fraction (%)	55 (50–60)	55 (50–60)	0.917
Blood lipids			
Triglycerides (mmol/l)	2.4 (1.2–2.7)	2.0 (1.5–3.3)	0.714
Total cholesterol (mmol/l)	4.2 (3.9–4.7)	4.7 (4.0–5.1)	0.354
LDL (mmol/l)	2.6 (2.2–3.0)	2.8 (2.1–3.1)	0.779
HDL (mmol/l)	0.9 (0.8–1.0)	1.1 (0.9–1.3)	0.021

Data are expressed as median (interquartile range) or number (%), as appropriate

*Black African and other

*BMI*, body mass index; *ACEI*, angiotensin-converting enzyme inhibitor; *ARB*, angiotensin II receptor blocker; *LDL*, low-density lipoprotein; *HDL*, high-density lipoproteins

**Table 2:** Operative and post-operative patient characteristics

	Control group (*n* = 13)	Intralipid group (*n* = 13)	*P* value
Operative characteristics			
Cross-clamp time (min)	80 (69–86)	80 (71–109)	0.600
Number of bypass grafts	3 (3–3)	3 (3–3)	> 0.99
Ventricular fibrillation	2 (15%)	0 (0%)	0.140
Post-operative characteristics			
Mechanical ventilation time (hours)	16 (12–18)	15 (13–17)	0.909
ICU length of stay (hours)	45 (42–68)	46 (44–70)	0.772
Hospital length of stay (days)	7 (6–8)	7 (7–7)	0.861
Serious adverse events			
Death	0 (0%)	1 (8%)	> 0.99
Other	5 (38%)	4 (31%)	> 0.99

Data are expressed as median (interquartile range) or number (%), as appropriate

*ICU*, intensive care unit

**Fig. 3:**
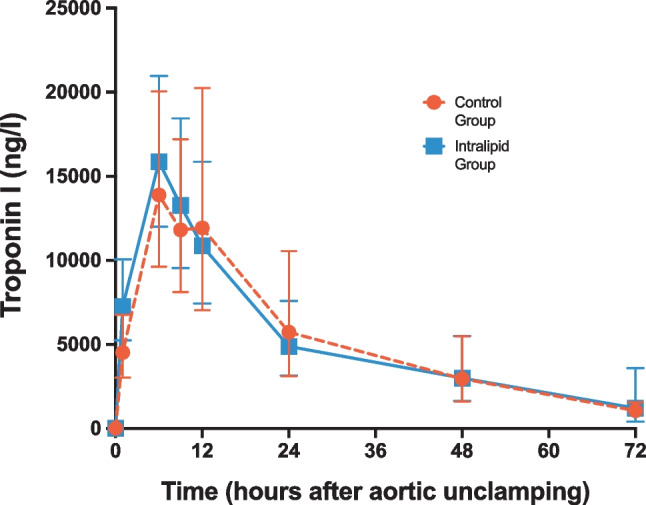
Assessment of myocardial injury (cardiac troponin I). Geometric means and 95% confidence intervals of cardiac troponin I levels in blood are presented for the Control group (orange circles) and the Intralipid group (blue squares) at baseline and 1, 6, 9, 12, 24, 48, and 72 h after unclamping the aorta

**Fig. 4:**
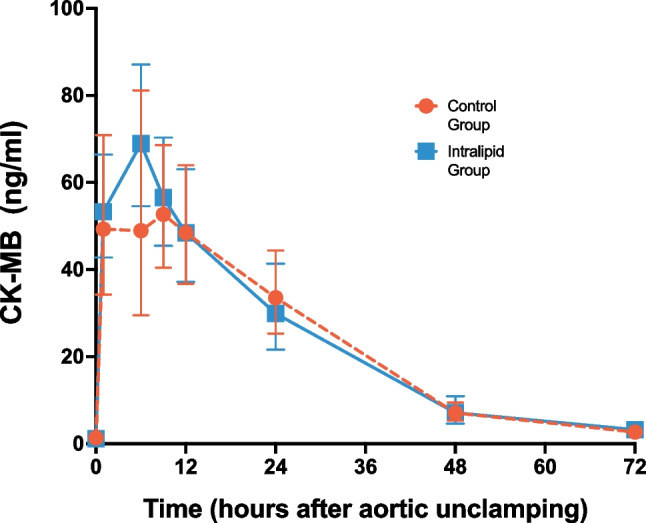
Assessment of myocardial injury (CK-MB). Geometric means and 95% confidence intervals of CK-MB levels in blood are presented for the Control group (orange circles) and the Intralipid group (blue squares) at baseline and 1, 6, 9, 12, 24, 48, and 72 h after unclamping the aorta

As shown in Fig. 3, the 72 h AUC of cTnI after aortic unclamping did not significantly (*P* = 0.804) differ between the Intralipid group (487552 ± 115724 arbitrary units (AU)) and the Control group (546430 ± 205518 AU). The 72 h AUC of CK-MB was 1916 AU in the Intralipid group versus 1889 AU in the Control group (*P* = 0.956) (Fig. 4). In addition, there was no significant difference in both cTnI and CK-MB levels between the Intralipid and Control groups (*P* = 0.985 and *P* = 0.815, respectively, by repeated measures).

At 24 h after surgery, CRP was 134 (90–171) mg/l in the Control group and 126 (114–139) mg/l in the Intralipid group (*P* = 0.809). Other post-operative endpoints including duration of invasive mechanical ventilation, ICU length of stay, hospital length of stay, and number of serious adverse events were similar in both groups (Table 2).

## Discussion

In the CREW-I pilot, double-blinded, randomized, monocentric trial, Intralipid 20% administered upon reperfusion during CABG on CPB in patients from South Africa, did not prevent IRI assessed by release in cTnI within 72 h after surgery, compared to placebo.

Four small pilot-randomized control trials (RCT), that included each between 30 and 73 patients, have previously investigated the cardioprotective potential of Intralipid in cardiac surgery [8–11]. All of them reported the feasibility and safety of Intralipid administration in patients undergoing either valve replacement surgery or off-pump CABG surgery. The present study, showing no side effects related to the Intralipid administration, extended these findings to patients undergoing CABG on CPB. Regarding the efficacy of Intralipid in reducing IRI during cardiac surgery, no conclusions can currently be made with certainty. Indeed, if we include the present CREW-I trial, only 3 out of 5 RCT were positive. The 2 RCT that included patients undergoing CABG reported a decrease in markers of myocardial injury with Intralipid (Fresenius Kabi) [10, 11]. However, it is difficult to compare these results with our findings as surgery was performed without CBP (i.e. off-pump), which may induce different mechanisms of IRI [17]. In the 2 other trials, cardiac surgery was performed under CPB but did not include patients undergoing CABG [8, 9]. The first one, published in 2017, showed a 32% decrease in the AUC of cTnT with 2 ml/kg 20% Intralipid (SINO SWED Pharmaceutical, Jiangsu, China) administered 10 min prior to cross-unclamping in patients undergoing aortic or mitral valve surgery [8]. In contrast, the next year, the same group of investigators did not observe any benefits of Intralipid when valve replacement surgery was performed with concomitant radiofrequency ablation [9]. Altogether, the discrepancy between studies suggests that Intralipid is not beneficial for all patients undergoing cardiac surgery. Ongoing larger RCT will determine whether Intralipid can limit IRI in cardiac surgery patients on CPB [18].

Several factors may explain the neutral results of the CREW-I trial in comparison with the RCT that demonstrated cardioprotection with Intralipid. First, this was the first time that Intralipid was used to prevent IRI during cardiac surgery in a cohort of patients from Sub-Saharan Africa; previous positive RCT have been conducted in Asia (China and India) and North Africa [8–11]. Therefore, it is possible that the protective effects of Intralipid may depend on the type of population (e.g., ethnicity, socioeconomic status, medications) [19, 20]. However, the severity of IRI assessed in our study (using by cTnI for 72 h) was similar (including the peak of cTnI and the shape of the curve) to that of patients from Western Europe undergoing CABG on CPB [16]. Second, the drug that was tested (i.e., lipid emulsion) was not the same in all RCT. For instance, we used Intralipid made by Fresenius-Kabi in South Africa whereas lipid emulsion was provided by SINO SWED Pharmaceutical Corps in the two RCT conducted in China [8, 9]. As the formulation of lipid emulsion (especially percentage of cardioprotective palmitoylcarnitine) [6, 21] can significantly differ among manufacturers, it is possible that other formulations might have yield to different results.^3,21^ Nevertheless, we found that Intralipid used in the CREW-I trial was indeed cardioprotective in our ex-vivo model of IRI. Similarly, the doses of Intralipid (1.5 to 2 ml/kg) and timing of Intralipid administration (before CABG, 10 min or 3 min before unclamping the aorta) varied among RCT and these differences may have also contributed to the heterogeneity of the results. Third, protocol of anesthesia might have also influenced our results. Because Propofol contains lipids and presumably prevents protection afforded by remote ischemic conditioning, we used inhaled sedation (Isoflurane) to maintain anesthesia [22, 23]. It is unknown whether Isoflurane could have interfered with Intralipid for cardioprotection. Nevertheless, the fact that Intralipid was found to be protective in patients undergoing off-pump CABG who were anaesthetized with Isoflurane does not support this hypothesis [10, 11].

A better understanding of the cardioprotective signaling pathways that may protect against IRI is likely to enhance the design of suitable strategies that could protect the human heart [13, 24]. An experimental study in pregnant mice and rats convincingly demonstrated, using in vivo and ex vivo models of acute myocardial infarction, that Intralipid significantly limited infarct size via the activation of STAT3 [14]. However, it is important to keep in mind that both sex and pregnancy affect STAT3 signaling, and their findings may therefore not be generalized to the population used in our study [25, 26]. In an isolated heart model of IRI without myocardial infarction that better approximated the pathophysiology of cardiac surgery on CPB than a model of acute myocardial infarction, Lou et al. did not observe any difference in STAT3 activation between the Intralipid group and the control group although Intralipid significantly enhanced cardiac recovery after ischemia–reperfusion injury [6]. It would be interesting to assess the effects of Intralipid on STAT3 signaling in clinical trials in which Intralipid is cardioprotective.

This study has limitations. First, only 26 of the 30 patients initially planned were included in the final analysis. Recruitment of patients had to be stopped before completion because of the COVID-19 pandemic, which severely impacted our capability of conducting high-quality clinical research. Nevertheless, despite the well-balanced characteristics of the patients, we did not highlight any signal suggesting cardioprotection with Intralipid. Therefore, it is very unlikely that the inclusion of four or more patients would have changed the results. Second, we may have overestimated the effect of Intralipid on the reduction of myocardial IRI during cardiac surgery, as suggested by proof-of-concept clinical trials [8–11]. Indeed, we designed our trial before the publication of other clinical trials and thus calculated the sample size based on preclinical data [4–7] and on a randomized clinical trial in 24 well-selected patients undergoing CABG showing a significant reduction (> 50%) in the 72 h AUC of cTnI [16]. Third, the trial was conducted in a single center with most patients of sub-Saharan African origin, which may limit the conclusion of the findings. Fourth, we excluded individuals aged ≥ 65 years and those with comorbidities from our study, as these factors have been shown to alter the efficacy of cardioprotective interventions [15]. Despite our efforts to maximize the potential of observing cardioprotection with Intralipid in this trial, it remains unclear whether similar results would be obtained in patients with more comorbidities. Lastly, it is possible that the type of cardioplegia may affect the efficacy of Intralipid. Nonetheless, in a study in which Intralipid demonstrated cardioprotective properties, the investigators employed cold blood cardioplegia, similar to the method used in the present investigation [8].

In conclusion, Intralipid, given at the onset of reperfusion in patients undergoing CABG on CPB, was safe but did not limit myocardial IRI compared to placebo.

## Data Availability

Research data associated with this paper can be accessed by contacting the authors directly.

## Abbreviations

AUC: Area under the curve
CABG: Coronary artery bypass grafting (CABG)
CK-MB: Creatine kinase—myocardial band
CPB: Cardiopulmonary bypass
CREW-I: Cardiac REperfusion With Intralipid at reperfusion study
CRP: C-reactive protein
cTnI: Cardiac troponin I
HDL: High-density lipoprotein
ICU: Intensive care unit
IRI: Ischemia-reperfusion injury
LDL: Low-density lipoprotein
RCT: Randomized controlled trial
SAFE: Survivor Activating Factor Enhancement
STAT3: Signal Transducer and Activator of Transcription 3
